# A Novel Vibration Mode Testing Method for Cylindrical Resonators Based on Microphones

**DOI:** 10.3390/s150101954

**Published:** 2015-01-16

**Authors:** Yongmeng Zhang, Yulie Wu, Xuezhong Wu, Xiang Xi, Jianqiu Wang

**Affiliations:** College of Mechatronics Engineering and Automation, National University of Defense Technology, Changsha 410000, China; E-Mails: zymnudt@163.com (Y.Z.); ylwu_nudt@sina.com (Y.W.); fordada@126.com (X.X.); wjq19901122@163.com (J.W.)

**Keywords:** vibration mode testing, cylindrical resonator, vibratory cylinder gyroscope, MEMS microphone

## Abstract

Non-contact testing is an important method for the study of the vibrating characteristic of cylindrical resonators. For the vibratory cylinder gyroscope excited by piezo-electric electrodes, mode testing of the cylindrical resonator is difficult. In this paper, a novel vibration testing method for cylindrical resonators is proposed. This method uses a MEMS microphone, which has the characteristics of small size and accurate directivity, to measure the vibration of the cylindrical resonator. A testing system was established, then the system was used to measure the vibration mode of the resonator. The experimental results show that the orientation resolution of the node of the vibration mode is better than 0.1°. This method also has the advantages of low cost and easy operation. It can be used in vibration testing and provide accurate results, which is important for the study of the vibration mode and thermal stability of vibratory cylindrical gyroscopes.

## 1. Introduction

The vibratory cylinder gyroscope (VCG) is a type of solid-state wave gyroscope which senses the angular rate based on the inertia effect of the elastic wave in a cylindrical resonator [1]. Compared with the conventional spinning wheel gyroscope, the vibratory cylinder gyroscope is considerably more rugged, can be started up much more quickly, consumes much less power and has no bearings which could be susceptible to wear [2]. Compared to fiber optic and MEMS gyroscope technologies, the vibratory cylinder gyroscope has competitive accuracy characteristics with respect to the Fiber Optic Gyroscope (FOG), but costs less; besides, it has a higher accuracy compared to current MEMS gyroscopes due to its larger vibrating mass, with the additional advantage that it does not require expensive special equipment [3].

The key component of the vibratory cylinder gyroscope is the cylindrical resonator, which is excited and maintained in a kind of vibration mode with four antinodes [4]. The offset of the vibration mode of the cylindrical resonator is the main reason of the drift of the vibration cylinder gyroscope [5], thus it is important to research the stability of the vibration mode of the cylindrical resonator to improve the performance of vibratory cylinder gyroscopes.

The research concerning the vibration mode of the cylindrical resonator is extremely wide. The offset of the vibration mode is caused by the imperfections of the cylindrical resonators, such as the effects of manufacturing errors and material inhomogeneity [6]. Studies on the stability of axisymmetric forced vibrations of imperfect structures are reported [7]. Moreover, the standing wave vibration of the imperfect resonant shell for cylindrical gyroscopes was investigated [8].

For the sake of in depth research into the drift of vibratory cylinder gyroscopes, it is necessary to accurately measure the offset of the vibration mode of the cylindrical resonator. Laser vibrometers are used to measure the vibration of one spot on the surface of the resonator, then the mode shape of the resonator can be obtained by a spot scan method [9]. Pattern angle error *vs* pattern angle and output of the quadrature null loop *vs* pattern angle are measured by the electrostatic method [10].

The vibratory cylinder gyroscope is excited and sensed by piezoelectric electrodes, which are pasted on the bottom or the wall of the cylindrical resonator. It is difficult and time consuming to measure the distribution of the vibration with piezoelectric electrodes, as the piezoelectric electrodes should be repasted on different angular positions of the resonator which will affect the vibrating characteristics of the cylindrical resonator. Therefore, non-contact measurement is an important method for vibration testing. The current non-contact measurement technique is to use a laser vibrometer. However, laser vibrometers are expensive, difficult to manipulate and cannot operate continuously for a long time, and these disadvantages make the testing of the vibration mode time consuming and expensive.

This paper proposes a high-accuracy, low-cost vibration testing method for vibratory cylinder gyroscopes. This method uses a MEMS microphone, which has a micro size and accurate directivity, to measure the vibration of the cylindrical resonator. A testing system was established and used to measure the vibration mode of a cylindrical resonator. The experimental results show that the orientation resolution of the node of the vibration mode is better than 0.1°. Moreover, this method also has the advantages of low cost and easy operation.

## 2. Operation Principle of the Vibratory Cylinder Gyroscope

**Figure 1.**
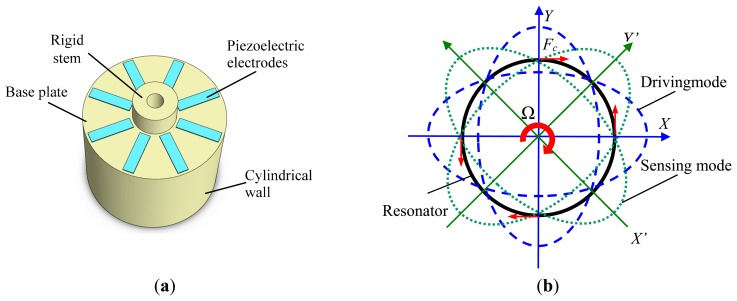
(**a**) Structure of the vibratory cylinder gyroscope; (**b**) Schematic of the operation principle.

The basic structure of the vibratory cylinder gyroscope is shown in Figure 1a. The cylindrical resonator comprises a rigid stem, a base plate and a cylindrical wall. Eight evenly distributed piezoelectric electrodes are pasted on the base plate and used to excite and sense the vibration of the resonator [5].

The operation principle of the vibratory cylinder gyroscope is illustrated in Figure 1b. By applying an alternating voltage to the two piezoelectric electrodes attached to the bottom in the X-axis, the resonator is excited into the driving mode in the *X*-*Y* axes direction. When the resonator rotates at an angular velocity Ω, a sensing vibration mode starts to appear due to Coriolis force vectors. The output voltage of the electrodes on the *X′*-*Y′* axes is proportional to the angular velocity Ω and can be detected by a readout circuit [11]. The geometry parameters of the resonator are shown in Table 1.

## 3. Vibration Mode Testing Based on the Mems Microphone

### 3.1. Testing Principle

As a matter of experience, the amplitude of the resonant shell can reach an order of micrometers, and the resonant frequency is on the order of kHz. For the resonator shown in Figure 1a, when the radius of the shell is 25.4 mm, and the height is 18 mm, the resonant frequency of the resonator will between 3 and 5 kHz, which is in the frequency range of human hearing and most microphones.

**Figure 2.**
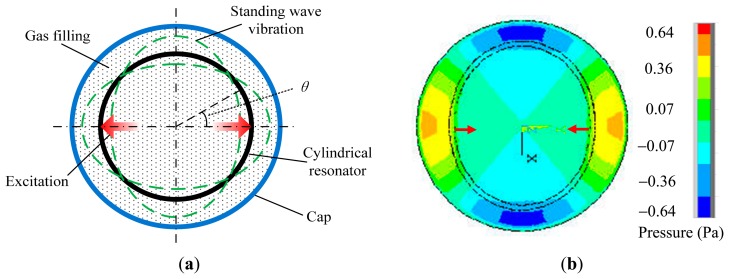
(**a**) Cylindrical resonator in a cap; (**b**) Acoustic pressure contour of the vibratory cylinder gyroscope.

According to the acoustic theory, in a fluid, the diffuse direction of the sound vibration is coincident with the vibrating direction of the medium particle. This kind of sound vibration process is called longitudinal wave. Thus, there are only longitudinal sound waves in the fluid. During the working process of the vibratory cylinder gyroscope, the cylindrical resonator vibrates in the form of a standing wave with four antinodes. The cylindrical resonator is covered by a cylindrical cap, and the cap is filled with a dry gas under atmospheric pressure, as shown in Figure 2a.

The radial displacement of the cylindrical resonator can be written as:

(1) $$w\left(\theta ,t\right)=\frac{F_{ex}}{K}Q\cos2\theta \cos\omega t$$

where *w* is the radial displacement of the resonator, *F_ex_* is the amplitude of the exciting force, *Q* is the quality factor of the resonator, *K* is the bending stiffness which can be calculated by $K=\frac{9\pi EI}{2R^{3}}$ (*E* is Young's modulus; *I* is the inertia moment of the resonator, *R* is the radius of the cylindrical resonator), *θ* is the angular position, *ω* is the eigenfrequency which can be calculated by $\omega =\frac{h}{R^{2}}\sqrt{\frac{3E}{5\rho_{r}\;\left(1-\mu^{2}\right)}}$ (*h*_1_ and *h*_2_ are the height and width of the resonator respectively) [12].

The dynamics equation of the sound wave is:

(2) $$\rho_{0}\frac{\partial \nu }{\partial t}=-\frac{\partial p}{\partial x}$$

Equation (2) shows that the amplitude of the acoustic pressure has a positive correlation with the vibrating velocity, which is proportional to the amplitude of the vibration, so the acoustic pressure of the antinodes is maximal, and the acoustic pressure of the nodes is minimum. The distribution of the acoustic pressure around the cylindrical resonator can be seen in reference [13].

In reality, it is usually difficult to obtain analytical solutions of the complicated acoustic equations, therefore the FEM software ANSYS is employed to analyze the acoustic pressure distribution of the cylinder gyroscope. The acoustic pressure contour of the vibratory cylinder gyroscope at the resonant frequency is obtained, as shown in Figure 2b [13]. It can be seen that the distribution of the acoustic pressure is closely related to the standing wave vibration of the resonator. In the radial direction, the acoustic pressure is symmetrical, and reaches its maximum value at the antinodes of the standing wave. In addition, the acoustic pressure between the cylindrical resonator and the cap is much larger than that inside the cylindrical resonator.

### 3.2. Testing System

**Figure 3.**
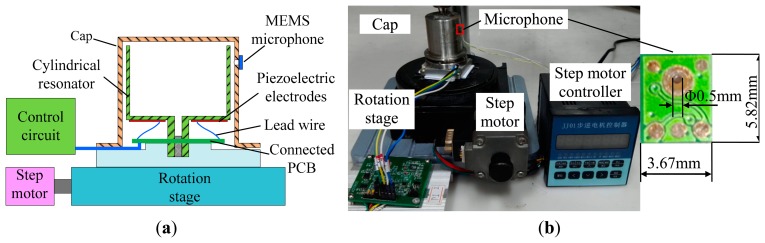
(**a**) Schematic of the testing system; (**b**) Photograph of the testing system.

As analyzed in Section 3.1, the distribution of the acoustic pressure is closely related to the standing wave vibration of the resonator, so we can use a microphone to measure the acoustic pressure around the resonator, and then the distribution of the acoustic pressure can be obtained. The testing system is illustrated as Figure 3a.

**Figure 4.**
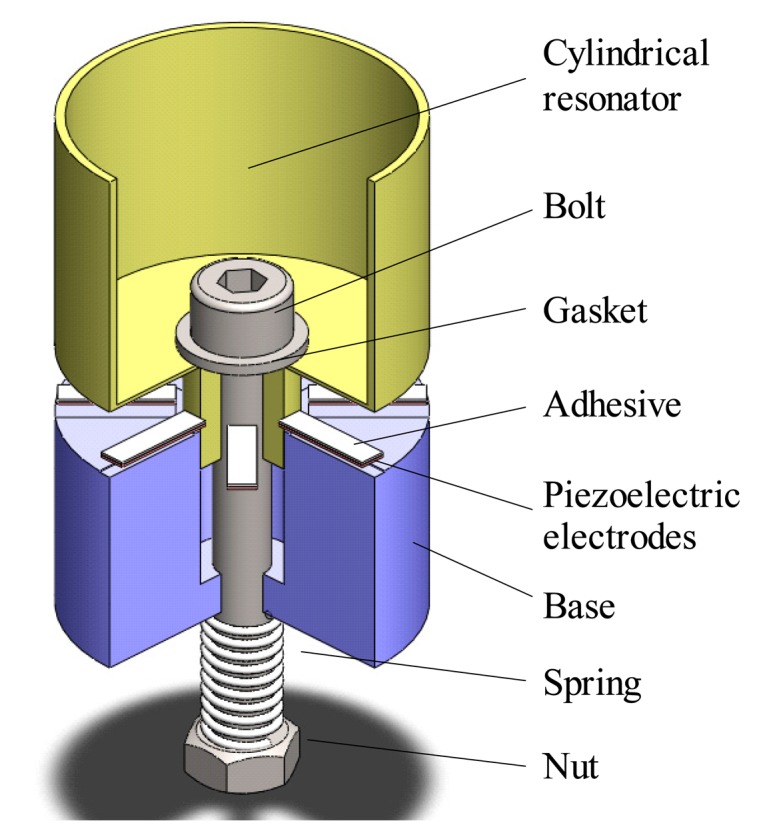
Schematic of the pasting of the piezoelectric electrodes.

The piezoelectric electrodes are pasted on the bottom of the resonator as shown in Figure 1a; here, a conductive adhesive is used. The process of the pasting the piezoelectric electrodes is shown in Figure 4. A base with eight grooves is designed to ensure the precise orientation of the electrodes. A nut and a spring are used to provide proper strength to the electrodes as the piezoelectric electrodes are thin and fragile. After pasting the electrodes, the resonator is dried in 120 °C for two hours, then the conductive adhesive is solidified. The type of the conductive adhesive used is Techbond-TB-2608.

In Figure 3, the cylindrical resonator is mounted on a rotation stage, which is driven by a step motor. There is a PCB connected below the resonator, and the piezoelectric electrodes are connected to the control circuit by lead wire and the connected PCB. The resonator is covered with a cap, and on the sidewall of the cap, there is a through hole, where the microphone is pasted. When the cylindrical resonator rotates with the rotation stage, the cap is immovable, so the microphone can measure the acoustic pressure around the resonator. The rotation angle of the rotation stage can be accurately controlled by the number of impulses sent to the stepping motor. Moreover, the cap and the cylindrical resonator are homocentric, so the reflected wave is symmetrical and will not influence the orientation of the antinodes and nodes of the vibration mode.

A photograph of the testing system is shown in Figure 3b. The microphone of the testing system is a model number MSMAS42Z produced by MEMSensing Microsystems Co. (Suzhou, China). It has a small size, high sensitivity, wide frequency range, high signal to noise ratio and low cost. The type of the step motor is Y09-42D3-6151, it rotates 1.8° with one pulse. The transmission ratio of the rotation stage is 180:1, so the rotation stage will rotate 0.01° with one pulse input to the step motor.

## 4. Experiments

### 4.1. The Comparison between the Piezoelectric Electrodes and the Microphone

**Figure 5.**
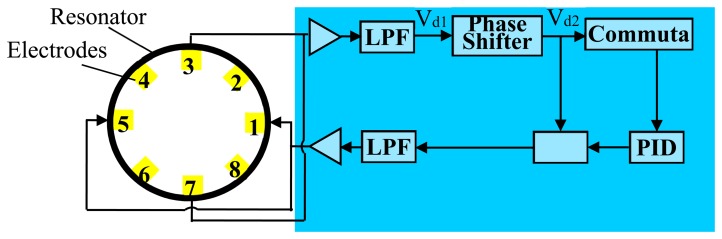
Electrical diagram of the driving circuit.

This experiment is designed to validate the linearity of the output of the MEMS microphone, and the experiment setting is shown in Figure 3. The resonator is driven by a control circuit shown in Figure 5, thus the cylindrical resonator is vibrating at the resonant frequency and the amplitude of the vibration can be regulated by the circuit. The whole control circuit of the vibratory cylinder gyroscope contains the driving loop, the sensing loop and the force rebalance loop [4], here we only use the driving control loop.

The amplitude of the vibration of the cylindrical resonator is of micron dimensions, which is very small, so the output of the piezoelectric electrode and the amplitude of the vibration have good linearity. The cylindrical resonator is driven by the electrodes 1 and 5, so the electrodes 1, 3, 5, and 7 are located at the antinodes of the vibration mode, the electrodes 2, 4, 6, and 8 are located at the nodes of the vibration mode. The driving circuit maintains the amplitude of the output of the electrodes 3 and 7 at a constant value. The amplitude of the vibration is measured by the microphone at the same time.

We regulate the orientation of the microphone by rotating the rotation stage, letting the microphone aim at the piezoelectric electrode 3. The piezoelectric electrode 3 is located at the antinode of the vibration mode, so when the output of the microphone reaches to a maximum, we can consider that electrode 3 is aligned with the microphone. We change the amplitude of the vibration of the cylindrical resonator by regulating the parameters of the driving circuit, recording the output of the piezoelectric electrodes 3 and the microphone under different amplitude conditions. The test results are listed in Table 2. If we use the microphone to measure the vibration of nodes 2 and 4, the linearity of the output of the microphone will be influenced by the alignment accuracy between the microphone and the electrodes, so we use the output of electrode 3 to study the linearity of the microphone.

**Figure 6.**
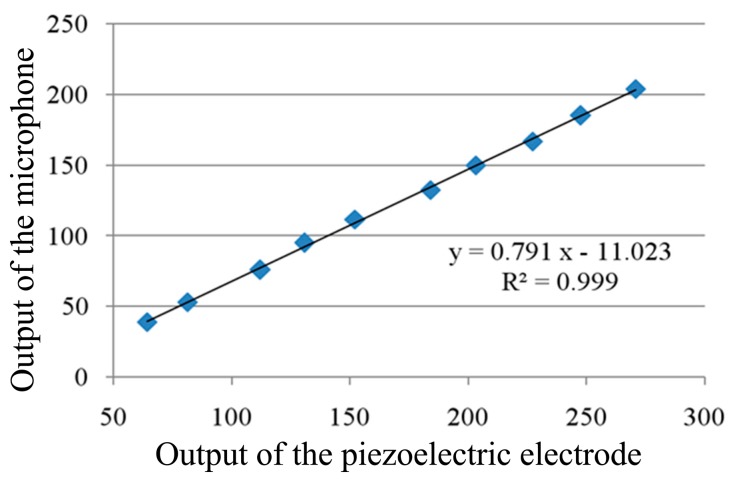
Testing results of the piezoelectric electrode and the microphone.

As shown in Figure 6, the horizontal axis is the output of the piezoelectric electrode and the vertical axis is the output of the microphone. After linear fitting of the data, the slope of the straight line is 0.791, and the correlation coefficient is 0.999, which is close to 1 and means there is good consistency between the output of the piezoelectric electrode and the microphone, so the output of the MEMS microphone also has a good linearity with the amplitude of the vibration.

### 4.2. Vibration Mode Testing

**Figure7.**
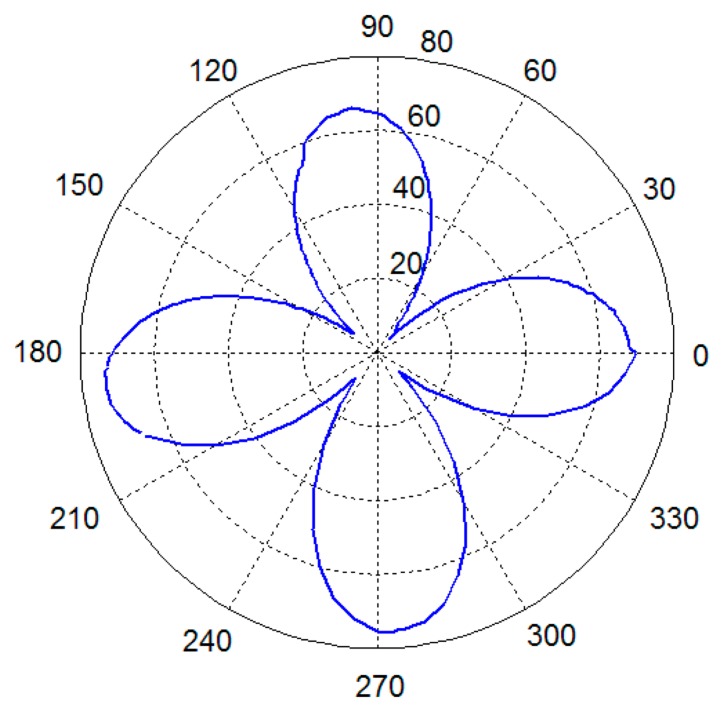
Testing results of the vibration mode of the cylindrical resonator.

**Figure 8.**
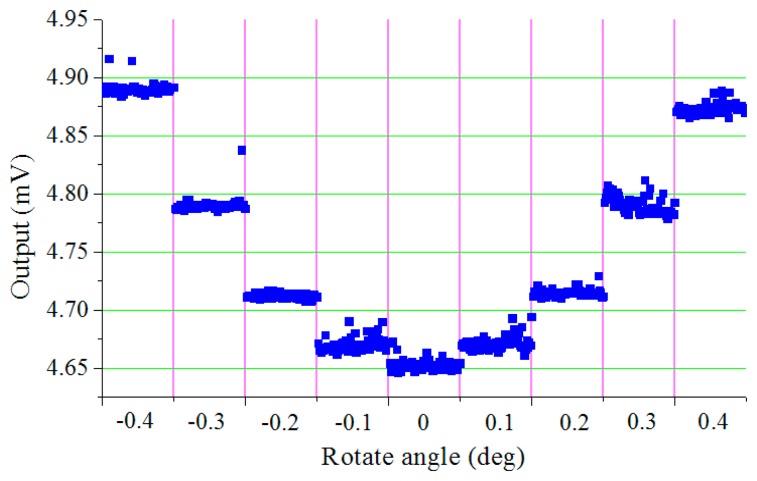
Testing the resolution of the system.

In this experiment, the vibration mode of the cylindrical resonator is measured and the measurement precision is analyzed.The acoustic pressure around the cylindrical resonator is measured every 5°, then the pattern shape can be obtained by curve fitting, and the testing results are shown in Figure 7, from which a typical four antinodes vibration mode can be obtained. In order to identify the orientation of the nodes accurately, the acoustic pressure near the nodes is measured every 0.1°. Sixty data points are sampled at each orientation, and the testing results is shown in Figure 8. It can be seen that the output of the microphone has an obvious change when the resonator rotates 0.1°, so the orientation resolution of the node is better than 0.1°. More accurate orientation of the node of the vibration mode can be obtained by data interpolation.

## 5. Conclusions

In this paper, a high-accuracy, low-cost vibration testing method for vibratory cylinder gyroscopes is proposed. Theoretical analysis shows that the distribution of the acoustic pressure is closely related to the standing wave vibration of the resonator, so this method uses a microphone to measure the acoustic pressure around the resonator, then the vibration mode can thus be obtained. A testing system is established in this paper, and the experimental results show that the testing resolution of the node of the vibration mode is better than 0.1°. The testing system can be used to measure the vibration mode offset of the cylindrical resonator at different temperatures, which is a significant feature to improve the thermal stability of vibratory cylinder gyroscopes.

## Figures and Tables

**Table 1.** Geometry parameters of the resonator.

**Parameter**	**Value**	**Parameter**	**Value**
Height of cylindrical wall	18 mm	Thickness of cylindrical wall	1 mm
Radius of cylindrical wall	25.4 mm	Thickness of base plate	0.3 mm
Height of rigid stem	10 mm	Radius of rigid stem	8 mm
Length of the piezoelectric electrode	8 mm	Width of the piezoelectric electrode	2 mm
Thickness of the piezoelectric electrode	0.2 mm		

**Table 2.** Testing results of the piezoelectric electrode and the microphone.

Driving voltage(mV)	409	548	820	1054	1275	1486	1700	1901	2173	2382
Output of the microphone(mV)	64.2	81.1	112.1	130.5	152.5	183.8	203.0	227.1	247.4	270.6
Output of the piezoelectric electrode(mV)	38.9	53.1	76.4	95.1	111.5	132.4	149.8	166.7	184.9	204.0
